# Answer to May 2024 Photo Quiz

**DOI:** 10.1128/jcm.01194-23

**Published:** 2024-05-08

**Authors:** Christopher C. Attaway, Erick Tobin, Daniel D. Rhoads

**Affiliations:** 1 Department of Laboratory Medicine, Cleveland Clinic, Cleveland, Ohio, USA; 2 Department of Pathology, Cleveland Clinic Lerner College of Medicine, Case Western Reserve University, Cleveland, Ohio, USA; 3 Infection Biology Program, Lerner Research Institute, Cleveland Clinic, Cleveland, Ohio, USA; Mayo Clinic Minnesota, Rochester, Minnesota, USA

**Keywords:** bacteriology, identification, blood culture, gram-positive bacteria, Streptococcus

## Abstract

Read the full article for the answer.

## ANSWER TO PHOTO QUIZ

The Gram stain reveals Gram-positive cocci in chains with some areas of over-decolorization, which is compatible with *Streptococcus* species. Nonhemolytic white colonies grew on the sheep blood agar. Interestingly, the organism is catalase-positive and cross-reacts weakly with Lancefield group B latex reagent. There was no identification from VITEK MS (bioMérieux); however, the isolate was identified as *Streptococcus halichoeri* by MALDI Biotyper (Bruker Daltonics Inc.). Antibiotic susceptibilities were performed via Sensititre Streptococcus STP6F AST Plate (Thermo Scientific) and interpreted by Clinical and Laboratory Standards Institute (CLSI) guidelines for β-hemolytic group *Streptococcus* species (CLSI M100-ED33:2023) due to the close relationship of *S. halichoeri* to pyogenic streptococci (1, 2); however, prior authors have utilized the guidelines for viridans group *Streptococcus* species (1, 3). The isolate was susceptible to penicillin G, ceftriaxone, clindamycin, and vancomycin. The patient was treated and improved on vancomycin.

*S. halichoeri* was originally isolated from grey seals (*Halichoerus grypus*) and proposed as a distinct species in 2004 (4). It has since been identified in fish as well as terrestrial and aquatic mammals and presents as a zoonotic infection, manifesting as cellulitis, in humans (5). The taxon is closely related to more commonly encountered large-colony streptococci including *S. agalactiae* and *S. dysgalactiae. S. halichoeri* was first described as causing human infection in 2014 in a post-operative empyema (1, 3, 4). Because of the catalase positivity and nonhemolytic colonies, *S. halichoeri* can be misidentified as *Staphylococcus* species; however, the chaining arrangement of the cocci on Gram stain is not compatible with staphylococci. Lancefield group B cross-reactivity further confuses the situation as this is not *S. agalactiae*, although the reactivity is weak compared to *S. agalactiae* (1). *S. halichoeri* can be discriminated from *S. agalactiae* by pyrrolidonyl arylamidase (PYR) positivity and hippurate hydrolysis negativity. *S. pseudoporcinus* also cross-reacts with Lancefield group B reagents, but this species is catalase-negative and beta-hemolytic, differentiating it from *S. halichoeri* (6). VITEK 2 (bioMérieux) could misidentify the organism as *S. suis* or *S. pyogenes* (1, 7). Definitive identification of *S. halichoeri* can be achieved using contemporary matrix-assisted laser desorption/ionization-time of flight (MALDI-TOF) analysis which has been shown to reliably identify *S. halichoeri* in several studies using Bruker MALDI Biotyper non-clinically validated library (*S. halichoeri* is absent in the bioMérieux Vitek MS databases, clinically or non-clinically validated) (1, 7). 16S rRNA sequencing can be used; however, some laboratories reported variable success with 16S sequencing (4, 7). *S. halichoeri* is susceptible to many antibiotic classes including beta-lactams as is typical of large-colony streptococci, though some studies have shown isolates that were resistant or nonsusceptible to erythromycin, clindamycin, doxycycline, or tetracycline (1, 2, 7).
